# Improved Mechanical Performances of Hastelloy C276 Composite Coatings Reinforced with SiC by Laser Cladding

**DOI:** 10.3390/nano15010018

**Published:** 2024-12-26

**Authors:** Yuqing Tang, Zheng Lu, Xuan Zhang, Xihuai Wang, Shengbin Zhao, Mingdi Wang

**Affiliations:** School of Mechanical and Electrical Engineering, Soochow University, Suzhou 215137, China; 20224229016@stu.suda.edu.cn (Y.T.); 20205229039@stu.suda.edu.cn (Z.L.); 20224229033@stu.suda.edu.cn (X.Z.); 20235229101@stu.suda.edu.cn (X.W.)

**Keywords:** laser cladding, alloy powder, wear resistance, corrosion resistance

## Abstract

Composite coatings reinforced with varying mass fractions of SiC particles were successfully fabricated on 316 stainless steel substrates via laser cladding. The phase compositions, elemental distribution, microstructural characteristics, hardness, wear resistance and corrosion resistance of the composite coatings were analyzed using X-ray diffraction (XRD), scanning electron microscopy (SEM), energy-dispersive X-ray spectroscopy (EDS), Vickers hardness testing, friction-wear testing and electrochemical methods. The coatings have no obvious pores, cracks or other defects. The phase compositions of the Hastelloy C276 coating includes γ-(Ni, Fe), Ni_2_C, M_6_C, M_2_(C, N) and M_23_C_6_. SiC addition resulted in the formation of high-hardness phases, such as Cr_3_Si and S_5_C_3_, with their peak intensity increasing with SiC content. The dendrites extend from the bonding zone towards the top of the coatings, and the crystal direction diffuses from the bottom to each area. Compared with the dendritic crystals formed at the bottom, the microstructure at the top is mostly equiaxed crystals and cellular crystals with smaller volume. When SiC powder particles are present around the crystals, the microstructure of the cladding layer grows acicular crystals containing Si and C. These acicular crystals tend to extend away from the residual SiC powder particles, and the grain size in this region is smaller and more densely distributed. This indicates that both melted and unmelted SiC powder particles can contribute to refining the grain structure of the cladding layer. The optimal SiC addition was determined to be 9 wt%, yielding an average microhardness of 670.1 HV_0.5_, which is 3.05 times that of the substrate and 1.19 times that of the 0 wt% SiC coating. The wear resistance was significantly enhanced, reflected by a friction coefficient of 0.17 (43.59% of the substrate, 68% of 0 wt%) and a wear rate of 14.32 × 10^−6^ mm^3^N^−1^·m^−1^ (27.35% of the substrate, 40.74% of 0 wt%). The self-corrosion potential measured at 315 mV, with a self-corrosion current density of 6.884 × 10⁻^6^ A/cm^2^, and the electrochemical charge-transfer resistance was approximately 25 times that of the substrate and 1.26 times that of the 0 wt%. In this work, SiC-reinforced Hastelloy-SiC composite coating was studied, which provides a new solution to improve the hardness, wear resistance and corrosion resistance of 316L stainless steel.

## 1. Introduction

Laser cladding involves the application of a pre-selected cladding layer powder material onto the surface of a substrate material using various feeding methods. The powder materials are simultaneously melted along with a thin layer of the substrate surface through high-energy laser irradiation, which undergoes rapid solidification and cooling to form a low-dilution coating that establishes a robust metallurgical bond with the substrate surface [1,2,3]. The resulting cladded coatings significantly enhance the strength, friction and wear resistance, corrosion resistance, high-temperature resistance and oxidation resistance of the substrate surface. This improvement increases the reliability of the components during operation and effectively reduces additional maintenance times and costs associated with surface quality issues [4,5]. Zhang et al. [6] successfully prepared Hastelloy C276 coatings on martensitic stainless steel substrates via laser cladding, achieving coatings with high cavitation erosion resistance. The thickness of these coatings can reach 600 microns, and their microhardness measures 334.4 HV_0.05_, which is 33.2% higher than that of the substrates. In comparison to the substrates, the coatings demonstrate a significant enhancement in cavitation erosion resistance. Silicon carbide (SiC) hard phase powders are recognized for their high hardness, excellent wear resistance, impact resistance and corrosion resistance, and have been extensively studied by researchers [7,8]. Ning et al. [9] prepared Inconel 718/SiC composite coatings with varying SiC contents through laser cladding. As the SiC content increased, the microhardness of the cladding layer also improved gradually. Notably, the HV_0.5_ value of the coatings containing 10 wt% SiC increased by 170% compared to those without SiC, indicating that the formed carbides contribute positively to the enhancement of hardness. Furthermore, the wear resistance of the coatings can be enhanced by the addition of SiC reinforcement. Within the range of 3 wt% to 7 wt% SiC, an increase in SiC content leads to a decrease in the friction coefficient, while the wear trace gradually becomes shallower. The primary wear mechanism is abrasive wear, accompanied by oxidative wear, fatigue wear and adhesive wear. Zhi et al. [10] investigated the effect of laser cladding on the corrosion resistance of magnesium ZK60/SiC composites. The corrosion potential of the laser coatings was approximately 300 mV higher than that of the substrates, while the corrosion current was three orders of magnitude lower. Liu et al. [11] employed laser cladding (LC) to create Ni-SiC coatings on 45# steel substrates. The results indicate that the average microhardness values of Ni-30SiC and Ni-10SiC coatings are approximately 851.3 HV and 748.4 HV, respectively. Additionally, the corrosion current density of Ni-30SiC coatings is the lowest at 6.325 × 10^−6^ A/cm^2^. Tang et al. [12] prepared NiCrAl-SiC coating on AISI H13 steel by laser cladding (LC). The results show that the NiCrAl-SiC coating has excellent high-temperature wear resistance, and the COFs and wear rate decrease with the increase in SiC mass fraction. Li et al. [13] successfully fabricated SiC particle-reinforced CoCrFeNiCu high-entropy alloy (HEA) coating (CoCrFeNiCu (SiC) x, x = 0, 5, 10, 15 wt%) on 316L stainless steel by laser cladding technology. The results show that the microhardness, wear weight and friction coefficient of CoCrFeNiCu (SiC) 15 HEA coating are 568.4 HV, 0.9 mg and 0.35, respectively. With the increase in SiC content, the corrosion resistance of CoCrFeNiCu (SiC) xHEA coating is enhanced in 3.5% NaCl solution.

Current literature indicates that research on laser cladding of Hastelloy C276 alloy primarily focuses on the effects of single self-fluxing alloy powders on the properties of the cladding layer. However, to the best of our knowledge, there are currently no reports on the use of SiC hard phase particles to enhance the microstructure and properties of Hastelloy C276 alloy composite coatings. To improve the wear and corrosion resistance of the inner wall surface of the reaction kettle (316L) during the stirring process, as well as to strengthen the overall physical and chemical properties of this surface and reduce maintenance costs associated with damage and failure, we developed a composite powder material comprising Hastelloy C276 and SiC hard phase particles. This material demonstrates enhanced hardness, wear resistance and corrosion resistance. Our innovative approach aims to improve the overall mechanical properties of the Hastelloy C276 coating, particularly its corrosion resistance. Further studies have shown that varying the amounts of SiC hard phase particles significantly enhances the mechanical properties of the composite. Consequently, the combination of self-fluxing alloy and ceramic powder results in alloy powders with excellent bonding strength, high hardness, wear resistance and corrosion resistance following laser cladding, thereby enhancing the performance of the 316L substrate across various applications.

## 2. Experimental Procedures

### 2.1. Materials

A 200 mm × 100 mm × 15 mm piece of 316L stainless steel is utilized as the substrate. Prior to the laser cutting (LC) process, the substrate was ground using sandpaper with a grit size of 600, resulting in an average surface roughness (Ra) of approximately 0.4 μm. Subsequently, the surface was cleaned with absolute ethanol to eliminate any residual impurities, followed by drying and storage. The elemental composition of 316L is shown in Table 1.

**Figure 1:**
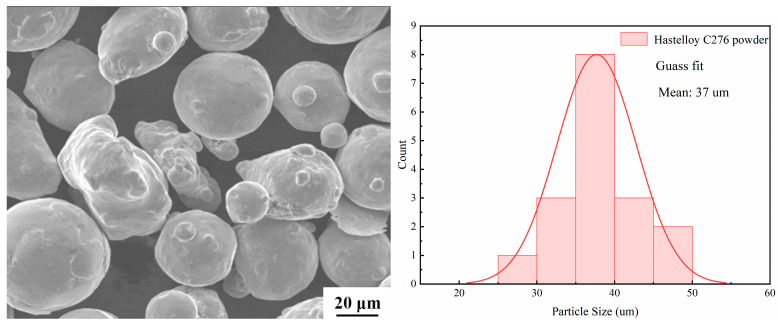
Hastelloy C276 powder morphology.

Hastelloy C276 was selected as the coating material. The density of Hastelloy C276 is 8.89 g/cm^3^. The SEM morphology of Hastelloy C276 is presented in Figure 1, while its elemental composition is detailed in Table 2 below. This material contains silicon and chromium, which have been demonstrated to exhibit excellent deoxidation and slagging performance. Furthermore, the inclusion of these elements has been shown to enhance the wetting and bonding capabilities of the molten metal powder to the substrate, reduce the oxygen content of the clad layer, minimize the porosity within the coating layer and improve the overall performance of the coating process [14]. To enhance the friction and wear resistance, as well as the corrosion resistance of the Hastelloy C276 cladding layer, varying amounts of SiC hard phase are incorporated into the cladding. The SiC hard phase is chemically stable, possesses a low coefficient of thermal expansion (3.80~5.12 × 10^−6^ °C^−1^), exhibits excellent resistance to friction and wear and has a very high hardness, with Vickers hardness ranging from approximately 2628 to 2946 HV (around 9.0–9.5) [15], second only to diamond, along with remarkable corrosion resistance. Its density and melting point are 3.17~3.47 g/cm^3^ and 2830 °C. These attributes significantly improve the physical and chemical properties of the Hastelloy C276 cladding layer. Consequently, the incorporation of SiC into the cladding layer markedly enhances the overall physical properties of the Hastelloy C276-SiC composite.

**Figure 2:**
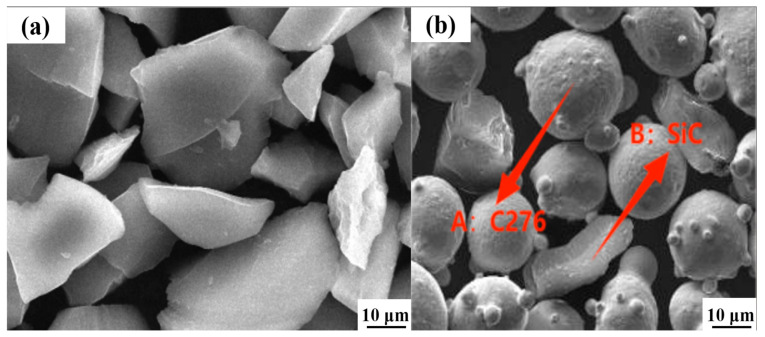
Powder morphology. (**a**) SiC powder; (**b**) mixed powder.

The Hastelloy C276 powder was weighed using an electronic balance, and silicon carbide (SiC) hard phases were added at mass fractions of 0%, 3%, 5%, 7%, 9% and 11%. These additions were incorporated into the Hastelloy C276 powder to prepare Hastelloy C276-SiC powder with varying SiC concentrations. To ensure uniformity in the powder mixture, the SiC powder and Hastelloy C276 were subjected to ball milling in a planetary ball mill for a duration of two hours. The scanning electron microscopy (SEM) morphology of the silicon carbide (SiC) hard phase is shown in Figure 2 below. As shown in the figure, the particle sizes of the SiC and C276 powders are comparable. SiC presents an irregular shape with an average size of 35 um.

### 2.2. Material Characterization and Property Tests

**Figure 3:**
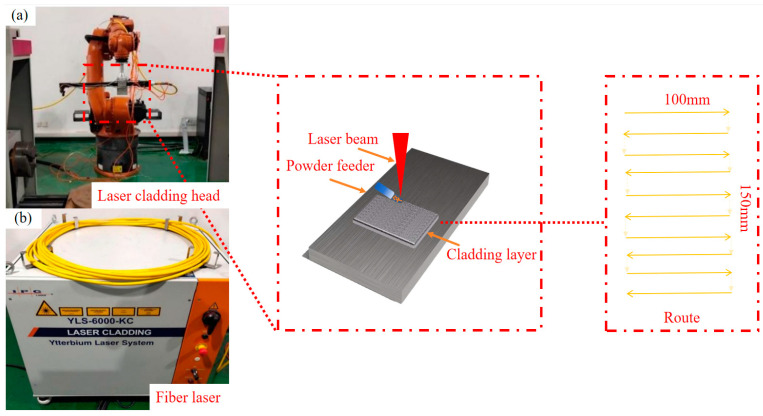
Schematic diagram of laser cladding process. (**a**) Laser cladding head; (**b**) Fiber laser.

The laser cladding system utilized in this experiment primarily consists of a three-dimensional motion platform, a wide-spot cladding head mounted on a robotic arm, a powder feeder, a water-cooling system, a high-power fiber laser and a gas-delivery system. As shown in Figure 3, the powder is precisely delivered by gas to the center of the melt pool from the powder feed port of the cladding head, where it rapidly solidifies on the surface of the substrate from its molten state.

The optimal process parameters were established as follows: a laser power of 3000 W, a powder-feeding speed of 10 g/min, a scanning speed of 6 mm/s, a lens-protection air pressure of 1.5 MPa and a powder-feeding gas flow rate of 4 L/h. These parameters were employed to conduct a single-layer laser melting processing test. The melted layers were subsequently cut into 10 mm × 10 mm × 10 mm cubes using a linear cutting machine, and then ground and polished to remove surface impurities. To minimize the impact of surface irregularities on diffraction results, a diffraction angle range of 35° to 100° was selected. The data obtained were imported into Jade 6.5 software for the processing of patterns and diffraction peaks. The generated phase diagram was qualitatively analyzed against standard reference cards to identify the surface phases of the tested object. The metallographic structure of the specimen’s cross-section was examined post-corrosion using an MX4R metallographic optical microscope (Ningbo Shunyu Instrument Co., Ltd., Ningbo, China). Surface morphologies were observed and photographed with a Zeiss EVO25 scanning electron microscope (Jena, Germany), while elemental analyses of point, line and surface areas were conducted on minute regions. The hardness of the specimens was measured using a Vickers microhardness tester, ensuring consistent load conditions and testing duration. Friction and wear tests were performed with an SFT-2M pin-disk friction and wear tester (Lanzhou Zhongke Kaihua Technology Development Co., Ltd., Lanzhou, China), utilizing parameters set at a load of 25 N, a friction duration of 45 min, a sample rotational speed of 300 r/min and a grinding ball made of Si_3_N_4_ with a diameter of 10 mm, recognized for its light weight, strength and hardness [16,17]. Tafel testing and AC impedance time potential measurements were conducted using a three-electrode workstation with a corrosion solution consisting of 3.5 wt% NaCl [18]. The AC impedance scanning frequency varied from 10^−2^ Hz to 10^5^ Hz, while the voltage was maintained at the steady-state open-circuit potential of ±5 mV. The spectral curves obtained were fitted and analyzed using electrochemical software, which facilitated the calculation of specific component parameters through a simulated equivalent circuit. Subsequently, the cyclic dynamic potential polarization curve of the working electrode was evaluated, from which the corrosion potential and corrosion current density were derived [19,20].

## 3. Results and Discussion

### 3.1. Phase Compositions

**Figure 4:**
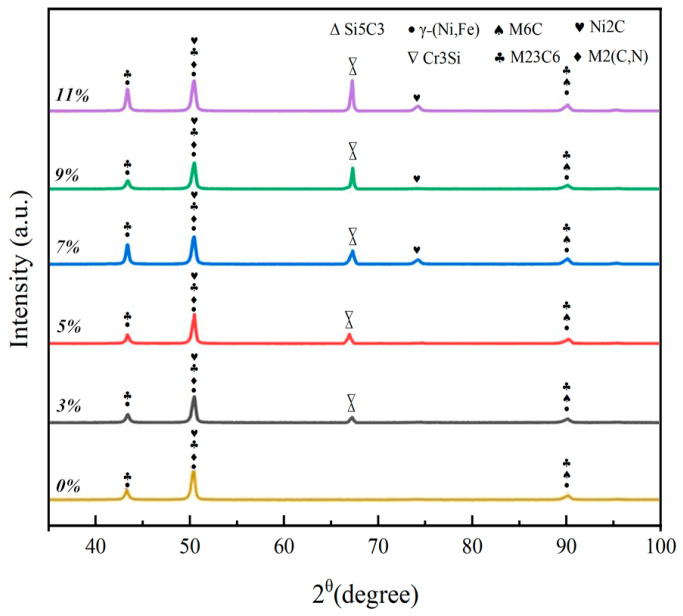
XRD pattern of Hastelloy C276-SiC cladding layer.

Figure 4 shows the X-ray diffraction (XRD) analysis of the cladded coatings of Hastelloy C276-SiC. The results indicate that the clad layer comprises several phases, including γ-(Ni, Fe), Ni2C, M6C, M2(C, N) and M23C6, with the predominant diffraction peak corresponding to the γ-(Ni, Fe) solid solution, where M primarily consists of Cr, Mo, Fe and Ni elements. This composition arises from the interaction of the laser beam with the substrates; as the laser heat is absorbed, the temperature in the melt pool increases, leading to convective phenomena that facilitate elemental interchange, thereby stabilizing the composition of the melt pool. The primary phase of the molten layers is identified as the γ-(Ni, Fe) solid solution. Notably, the M6C and M23C6 carbide phases contribute to enhancing the strength and hardness of the molten layers, while also improving their corrosion resistance and the density of their microstructure. Furthermore, the M2(C, N) phase, primarily composed of vanadium, promotes the formation of a diffuse distribution of fine particles through micro-alloying reinforcement. Additionally, the presence of the γ-(Ni, Fe) solid solution serves to encapsulate and support the carbide phases, thereby enhancing the stability and overall uniformity of the microstructure. This interaction also improves the plastic toughness of the cladded coatings, reducing the likelihood of defects in alloy hardness which could lead to surface cracks due to excessive nickel content. Following the incorporation of SiC-enhanced phases, high-hardness phases such as Cr3Si and Si5C3 were observed in the cladded coatings, with the peak intensities of these phases increasing progressively with the mass fraction of SiC. The addition of SiC will enhance the stability of decomposed carbon, resulting in the formation of more stable carbon-containing compounds with trace elements such as Si, Fe and Cr in Hastelloy C276. This modification can effectively improve the strength and hardness of the cladded coatings. Furthermore, the presence of various carbides within the eutectic structure ensures superior mechanical properties, thereby enhancing the hardness and wear resistance of the cladded coatings [21].

### 3.2. Microstructural Characteristics and Evolution

**Figure 5:**
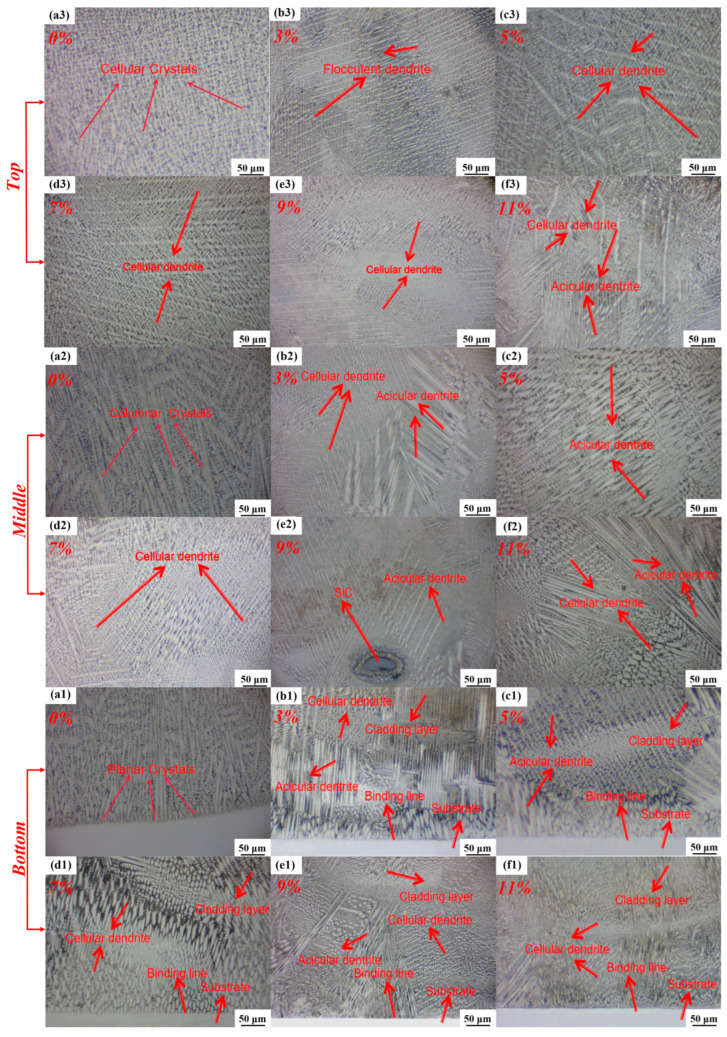
Metallographic diagram of Hastelloy C276-SiC cladding layers. (**a1**–**a3**) 0 wt% SiC; (**b1**–**b3**) 3 wt% SiC; (**c1**–**c3**) 5 wt% SiC; (**d1**–**d3**) 7 wt% SiC; (**e1**–**e3**) 9 wt% SiC; (**f1**–**f3**) 11 wt% SiC.

As shown in Figure 5, the cladding area is divided into top, middle and bottom. The top is the uppermost area of the cladding, the middle is the middle position of the cladding and the bottom is the lowermost area of the cladding. The metallographic microstructure images of the fusion zones at three different locations of the cladding layers of Hastelloy C276 and C276-SiC illustrate the bonding interface with the 316L substrate. Improper selection of the scanning speed, laser power and powder-feeding rate can adversely affect the laser energy and heat input. Excessive laser energy and heat input may cause the morphology of the bonding zone to exhibit a wavy contour due to elevated temperatures. Conversely, insufficient laser energy and heat input can lead to incomplete melting of the powder, resulting in residual powder that causes defects such as unbonded powder and porosity within the cladded coating. These defects severely impact the surface quality [22]. As indicated in Figure 5(a1–f1), the overall melt layer exhibits no significant porosity, cracks or spatter, resulting in a relatively smooth surface with a distinct metallic luster. The bonding region between the cladded coating and the substrate shows a uniform, continuous and clearly defined bonding line, demonstrating that the Hastelloy C276-SiC cladded coatings form a robust metallurgical bond with the 316L substrates, thereby ensuring a strong connection even under high operational stress conditions [23].

The metallographic microstructure images of the cladding layer with 0 wt% SiC, as shown in Figure 5(a1–a3), demonstrate that the microstructure predominantly comprises flocculent dendrites, columnar crystals, equiaxed crystals, cellular crystals and inter-dendritic regions. Notably, the majority of the crystal structures near the bonding zone between the substrate and the cladding layer are significantly larger than those located farther from this zone. This observed phenomenon can be attributed to the laser cladding process, during which the metal powder alloy attains a molten state at the center of the melt pool. The portion in contact with the substrate cools first, resulting in convection and diffusion phenomena between the bonding zone and the substrate. Consequently, the temperature gradient decreases from the bonding zone to the top of the cladding layer, which leads to a considerably faster growth rate of crystallites in comparison to the rate of nucleation.

As shown in Figure 5(b1–f1), the incorporation of SiC into the Hastelloy C276 powder leads to the formation of larger SiC particles, which predominantly accumulate at the bottom of the cladding layers. This phenomenon arises because, during the gravity-fed powder-delivery process, SiC attains higher kinetic energy, enabling it to reach the melt pool prior to the C276 powder. Following rapid solidification and cooling, these particles tend to cluster in the bottom region of the cladding layers.

The metallographic examination of the cladding layers, conducted from bottom to top, reveals that the overall microstructural appearance aligns with that of the 0 wt% SiC cladding layer, following a temperature gradient pattern. Flocculent dendrites extend from the bonding zone towards the top of the cladding layers, with crystal orientations dispersing from the bottom to various regions. In contrast to the flocculent dendrites observed at the bottom, the microstructure at the top primarily consists of smaller equiaxed grains and cellular dendrites. In addition to the crystallization patterns consistent with the 0 wt% SiC cladding layer, the presence of SiC powder particles surrounding the crystals promotes the growth of acicular dendrites containing Si and C, as illustrated in Figure 5(e2). These acicular dendrites tend to extend away from the residual SiC particles, resulting in a smaller and more densely distributed grain volume in this region. This observation indicates that both melted and unmelted SiC particles contribute to the refinement of the microstructural grains in the cladding layer, thereby enhancing its macro-level properties, such as strength, hardness and wear resistance.

As shown in Figure 5(f1–f3), when the SiC powder content exceeds 10%, the presence of equiaxed and cellular structures increases, exhibiting a fish-bone-like shape that is uniformly distributed throughout the cladding layer. XRD phase analysis indicates that these structures predominantly consist of silicides and carbides. However, when the SiC powder content reaches 11%, the larger size of the SiC particles significantly impedes the normal growth of dendrites. This results in the independent clustering of coarse, hard primary phases within the cladding layer, leading to a notable reduction in the density of the microstructural organization.

**Figure 6:**
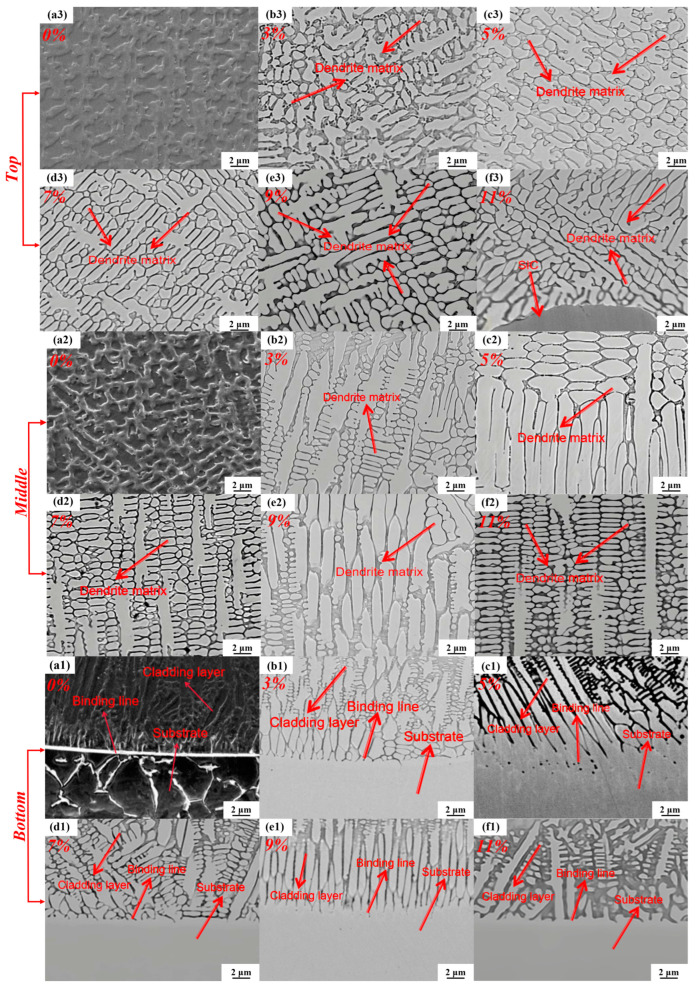
SEM images of Hastelloy C276-SiC cladding layers. (**a1**–**a3**) 0 wt% SiC; (**b1**–**b3**) 3 wt% SiC; (**c1**–**c3**) 5 wt% SiC; (**d1**–**d3**) 7 wt% SiC; (**e1**–**e3**) 9 wt% SiC; (**f1**–**f3**) 11 wt% SiC.

As shown in Figure 6, the SEM images of the bonding interface between the Hastelloy C276-SiC cladding layers and the 316L substrates reveal significant differences in microstructural sizes across the three regions. In Figure 6(a1–a3), the lower region depicts the bonding area between the 0 wt% SiC cladding layer and the 316L substrate. Here, the dendritic matrix primarily consists of large dendrites and columnar grains.

The temperature gradient in the central region of the cladding layer is less pronounced than that in the bonding area, where a greater degree of supercooling enhances the solidification rate. Consequently, the temperature gradients and solidification rates vary across different regions, exhibiting a general downward trend in the molten pool temperature gradients from the bonding zone to the top of the cladding layers. This variation results in differing nucleation states and growth rates for the dendritic matrix. Notably, the top of the cladding layers experiences the most significant temperature changes, leading to smaller grains compared to those in the central region. Additionally, the cellular structure at the top is denser than the acicular dendrite matrix found in the bonding and central regions [24]. Furthermore, distinct phases develop in various areas of the cladding layer as a result of the convective and diffusive interactions between the two materials at varying temperatures.

Elements such as Fe and B from the iron-based 316L substrate may diffuse into the bonding zone, resulting in distinct crystallization phases in the bonding area compared to those formed in the central and top regions. As illustrated in Figure 6(b–f), the microstructure of the Hastelloy C276-SiC cladding layers, which incorporate SiC powder, exhibits smaller overall grain sizes relative to the 0 wt% SiC cladding layer, with the original columnar grain structure transforming into smaller dendrites. Under optimal processing parameters for the C276-SiC cladding layers, SiC powder particles predominantly exhibit two morphologies. The first is partial dissolution diffusion, where the SiC particles retain their original shape with clear morphology, although the edges exhibit increased dissolution. This phenomenon occurs because high-melting-point trace elements such as Si, W and Cr absorb significant heat from the high-energy laser, thereby preventing the complete dissolution of the SiC particles at the base of the cladding layer. The second morphology is complete dissolution diffusion, in which SiC is entirely dissolved, resulting in a dendritic matrix. This process occurs as the SiC particles, propelled by argon gas into the melt pool, absorb substantial thermal energy from the laser, leading to dissolution diffusion.

The incorporation of SiC powder significantly inhibits dendrite growth and decreases grain size. As illustrated in Figure 6(f3), the microstructure surrounding the partially dissolved SiC particles reveals that the growth direction of the microcrystals extends outward from the particle surface, predominantly comprising dendritic matrices and cellular structures. This growth trend can be attributed to the substantial melting point difference between SiC and Hastelloy C276, which results in a pronounced temperature gradient. With an increase in SiC content, a majority of the partially dissolved SiC particles accumulate in the bonding zone due to their higher density compared to C276 powder, thereby contributing to a more compact microstructure within the cladding layers.

In the mid-region of the cladding layer with 11 wt% SiC, as illustrated in Figure 6(f2), the partially dissolved SiC particles inhibit crystal growth, resulting in a transformation of the large acicular and columnar dendrite matrix of pure Hastelloy C276 into smaller cellular structures. This observation suggests that the partially dissolved SiC powder can refine the microstructure of the cladding layer. Furthermore, the high SiC content contributes to dendrite matrix structures that are enriched with phases formed from trace elements such as Si, C, Cr, Ni and W carbides, leading to denser grain structures. However, an excessive accumulation of SiC may compromise the overall strength of the bonding interface, rendering the cladding layer more susceptible to spalling and cracking under severe wear conditions. Therefore, microstructural analysis indicates that the optimal physical properties of the Hastelloy C276-SiC cladding layer are achieved with a 9 wt% SiC addition.

**Figure 7:**
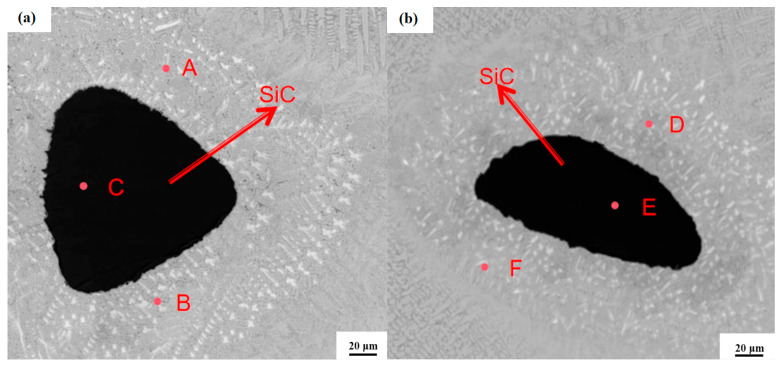
Microsolution diffusion SiC powder particles in the molten layer of 9 wt% SiC. (**a**) Microsolution diffusion SiC powder particles; (**b**) microsolution diffusion SiC powder particles.

The scanning electron microscopy (SEM) image depicting the microstructure surrounding the silicon carbide (SiC) particles within the molten layer containing 9 wt% SiC is shown in Figure 7 below. The results of the EDS point sweep analysis conducted around the SiC particles in this molten layer are summarized in Table 3 below. It is evident that the elemental composition and distribution of the cladding layer were modified following the incorporation of SiC. Consequently, a quantitative analysis of trace elements on the surface of the clad layer is essential to investigate the changes in elemental content and composition induced by the laser cladding process, as well as in the powder itself. Furthermore, the patterns of elemental distribution and changes in elemental content within the dendrite matrix require thorough analysis.

**Figure 8:**
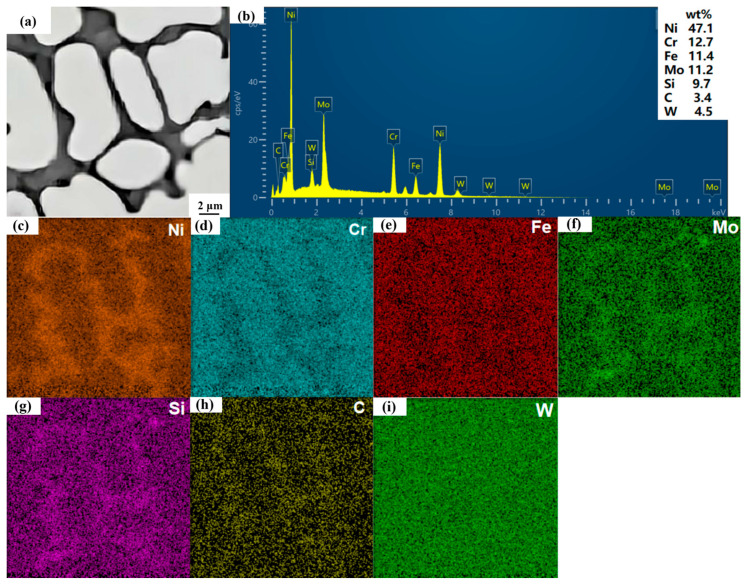
EDS mapping of 9 wt% SiC melting surface. (**a**) EDS mapping of the overall area; (**b**) overall elemental spectra of EDS mapping; (**c**–**i**) EDS mapping, elemental distribution of Ni, Cr, Fe, Si, C, W.

The EDS point sweep results for the six points A, B, C, D, E and F, as presented in Table 3, correspond to the six points indicated in Figure 7. From the surface sweep analysis results shown in (c) and (e) of Figure 8, it is evident that trace elements such as Ni and Fe are uniformly distributed across the surface of the molten layer. The XRD analysis reveals that the diffraction peak of C276-SiC is predominantly attributed to the γ-(Ni, Fe) solid solution. During the rapid cooling and solidification of the metal powder in the molten state, elements such as Ni, Cr and Mo combine with Fe to form a solid solution, which enhances the density of the microstructure of the clad layer, thereby improving its hardness, wear resistance and corrosion resistance [25].

The microsolution diffusion SiC powder particle diagram reveals that the surface of SiC particles is smooth and devoid of any discernible cracks or pits, thereby maintaining the original powder appearance. Furthermore, the results from the four-point sweep energy-dispersive X-ray spectroscopy (EDS) analysis at points A, B, D and F within the crystal structure indicate the presence of silicon (Si) and carbon (C). The X-ray diffraction (XRD) patterns of the clad layers containing SiC-added phases exhibit the emergence of new, high diffraction peaks corresponding to Si and carbide, in contrast to the XRD analysis of the clad layers composed of pure Hastelloy C276 [26]. This observation suggests that the SiC powder is melted by the high-temperature laser beam and subsequently undergoes rapid cooling and solidification in conjunction with the elements of Hastelloy C276, leading to the formation of various compounds that are widely distributed throughout the crystal structure.

Figure 7 are SEM images of micro-melting diffusion of SiC with two different shapes to better show the phenomenon of micro-melting diffusion. Moreover, the presence of SiC particles hinders the growth of the crystal structure, promoting a uniform distribution of Cr and Mo elements within the C276 powder’s dendrite matrix. A minimal amount of Cr in the dendrite matrix can significantly improve the resilience of the clad layer. Additionally, carbides formed at the grain boundaries, such as Cr23C6, can enhance the hardness and wear resistance of the material phase. Therefore, the incorporation of SiC can improve the overall physical properties of the clad layers [27].

### 3.3. Microhardness Analysis of Cladding Layer

**Figure 9:**
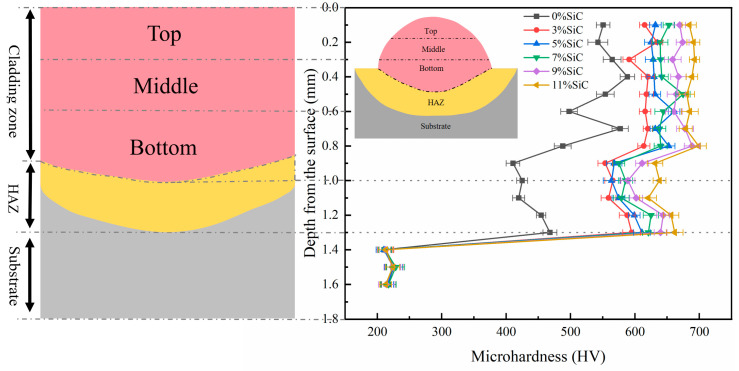
The microhardness distribution across the Hastelloy C276-SiC.

Figure 9 presents the comparative micro-Vickers hardness curves of the Hastelloy C276-SiC clad layers and the 316L substrates, measured in a direction normal to the bond line. The micro-Vickers hardness curve of the clad layers, processed under optimal parameters, exhibits four principal stages, which can be further categorized into four distinct segments: the clad layers, the alloying zone, the heat-affected zone and the 316L substrates. The overall trend reveals fluctuations. The average micro-Vickers hardness values for the Hastelloy C276-SiC clad layers with six SiC additions are 563.4 HV_0.5_, 616.1 HV_0.5_, 636.3 HV_0.5_, 646.8 HV_0.5_, 670.1 HV_0.5_ and 687.6 HV_0.5_, while the micro-Vickers hardness of the 316L substrate is 220.0 HV_0.5_. The micro-Vickers hardness of the clad layers with SiC addition is 2.56, 2.80, 2.89, 2.94, 3.05 and 3.13 times that of the substrate, with the maximum micro-Vickers hardness of the 11 wt% SiC clad layer reaching 699.6 HV_0.5_. The wear rates of the samples with 3 wt%, 5 wt%, 7 wt%, 9 wt% and 11 wt% SiC additions were found to be 1.09, 1.13, 1.15, 1.19 and 1.22 times that of the 0 wt% SiC sample.

Microscopic analysis indicates that Si and C elements originate from the dissolution of SiC powder within the clad layer, alongside Ni, Cr and other elements. Furthermore, the microsoluble diffusion of SiC powder particles can inhibit the growth of columnar crystals and other structures within the clad layers. This process also refines the grain size, thereby enhancing the density and uniformity of the microstructure. The observed gradual increase in hardness along the SiC addition curve supports this finding. The transformation trend in the three regions of the clad layers becomes increasingly smooth, with diminishing fluctuations, indicating that SiC addition enhances the overall microstructure of the clad layers, resulting in a uniform and dense structure. In conclusion, the addition of SiC powder effectively improves the overall hardness of the clad layer, promotes uniform distribution of the microstructure and ensures a seamless transition between the clad layers and the substrates, while stabilizing the hardness differences among the four regions [28].

### 3.4. Friction and Wear Behavior

**Figure 10:**
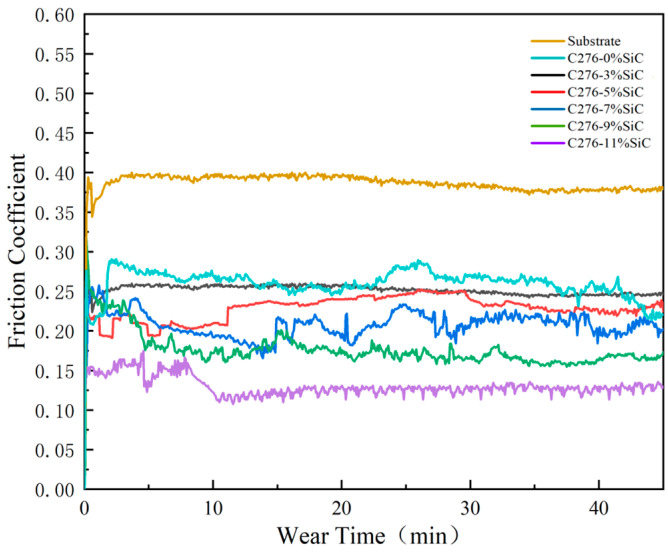
The friction coefficient curves of substrate and Hastelloy C276-SiC.

The trend of the friction coefficients at the interface between the Hastelloy C276-SiC cladding layers with varying SiC content and the 316L substrates is illustrated in Figure 10. The data indicate that as the SiC content increases, the friction coefficients of the cladding layers progressively decrease. The average friction coefficient of the 316L substrate is 0.39, whereas the average friction coefficients for the cladding layers with SiC additions of 0 wt%, 3 wt%, 5 wt%, 7 wt%, 9 wt% and 11 wt% are 0.25, 0.24, 0.22, 0.19, 0.17 and 0.12, respectively. This reduction in the friction coefficient can be attributed to several factors at the microstructural level. As shown in the SEM analysis in Figure 6, the microstructure of the 0 wt% SiC cladding layer primarily consists of small cellular crystals and dendritic structures, whose dense organization significantly enhances hardness and strength. XRD analysis results from Figure 4 indicate that the predominant phases in the 0 wt% cladding layer are hard phases and solid solution phases, which contribute to the overall strength and hardness of the surface structure. Consequently, the friction coefficient of the 0 wt% SiC cladding layer is lower than that of the substrate, leading to minimal fluctuations during testing and reduced material loss from the sample surface.

The incorporation of SiC further refines the microstructure of the Hastelloy C276-SiC cladding layers. The interaction between Si and C elements with Ni and Cr promotes the formation of new high-strength phases, such as Cr_3_Si and Si₅C_3_, which enhance microhardness and consequently reduce the average friction coefficient [29]. Furthermore, the partially dissolved SiC powder particles inhibit the growth of structures such as columnar crystals, contributing to grain size refinement and resulting in a more uniform and denser microstructure.

**Figure 11:**
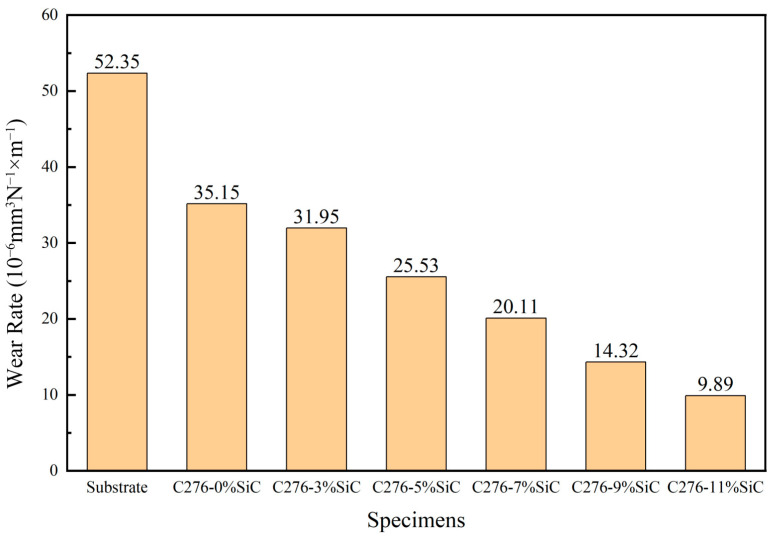
The wear volume of substrate and Hastelloy C276-SiC.

As shown in Figure 11, a comparison of the wear rates between the Hastelloy C276-SiC cladding layers with varying amounts of SiC addition and the 316L substrates was conducted under a load of 25 N for a duration of 45 min. The wear rates for the substrate and cladding layers with different SiC additions were measured at 52.35, 35.15, 31.95, 25.53, 20.11, 14.32 and 9.89 (×10^−6^ mm^3^ N^−1^·m^−1^), respectively. The wear rates of the six cladding layers corresponded to 67.10%, 61.03%, 48.77%, 38.41%, 27.35% and 18.90% of the wear rate of the 316L substrates. The wear rates for 3 wt%, 5 wt%, 7 wt%, 9 wt% and 11 wt% SiC additions correspond to 91%, 73%, 57%, 41% and 28% of the wear rate for the 0 wt% SiC sample, respectively. Under identical load and testing conditions, the wear rate of the 316L substrate was 52.35 × 10^−6^ mm^3^ N^−1^·m^−1^, while the wear rate of the 0 wt% SiC cladding layer was 35.15 × 10^−6^ mm^3^ N^−1^·m^−1^, representing only 67.1% of the substrate’s wear rate. This difference can primarily be attributed to the microelements Ni, Cr, Si and V in the 0 wt% SiC cladding layer, which crystallize into high-hardness and high-strength phases during the rapid solidification process in the cladding pool. Furthermore, under optimal processing parameters, the microstructural organization of the cladding layer is uniform and dense. The eutectics formed within the cladding layer enhance its hardness and strength, as indicated by the XRD analysis in Figure 4, where the Ni element in the γ-(Ni, Fe) solid solution plays a significant role in solid-solution strengthening. Additionally, the generated Cr, Mo and V carbides improve crystal strength and inter-phase lubrication, significantly enhancing the surface hardness and resistance to plastic deformation of the cladding layer, thereby increasing its wear resistance.

When Hastelloy C276 powder undergoes high-energy laser irradiation, it melts, and during the subsequent rapid solidification process, the cooling rate of the upper cladding layer exceeds that of the bonding region. This disparity results in significant grain refinement, which enhances hardness, strength and wear resistance compared to the substrate. It can be concluded that an increased addition of SiC in the laser cladding powder allows the microsoluble diffused SiC particles to impede dendrite growth during the rapid solidification process, transforming larger columnar and dendritic structures into smaller cellular crystals. Furthermore, the trace elements Si and C react with Ni and Cr to form new high-strength phases, such as Cr_3_Si and Si₅C_3_, which are uniformly distributed throughout the cladding layer. This distribution results in increased hardness and improved wear resistance relative to the substrate [30].

**Figure 12:**
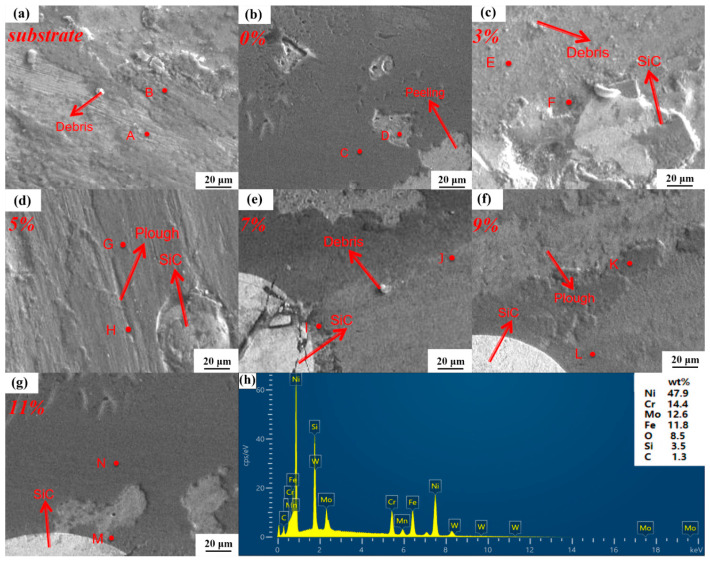
The SEM images of the Hastelloy C276-SiC cladded coating after friction wear. (**a**) Substrate; (**b**) 0 wt% SiC; (**c**) 3 wt% SiC; (**d**) 5 wt% SiC; (**e**) 7 wt% SiC; (**f**) 9 wt% SiC; (**g**) 11 wt% SiC. (**h**) EDS analysis results of worn surface (9 wt% SiC).

Figure 12h shows the findings from the comprehensive elemental surface sweep analysis conducted after the friction wear test of the 9 wt% SiC fused cladded coating. Table 4 below displays the results of the elemental point sweep analysis at the fourteen points marked A-N in Figure 12. As indicated in the table, oxygen is detected in the wear marks and furrow wear of each specimen following the 45-min friction wear test. The frictional wear of the cladded coating is primarily attributed to oxidation and mechanical wear. Initial oxidation wear is less severe than subsequent adhesive wear due to the protective nature of the oxide film. This oxide film, formed during oxidation wear, effectively reduces adhesion during adhesive wear under the combined effects of friction and wear, thereby enhancing the service life of the grinding ball. Consequently, when the proportion of oxidation wear in the overall wear process is higher, the presence of oxygen in the pits and scratches identified during the point sweep analysis also increases correspondingly. The results of the point sweep analysis presented in Table 4 clearly demonstrate that the proportion of oxygen in the molten layer of 3 wt% SiC is the highest. Residual high-hardness SiC powder particles are uniformly distributed within the melt layer, which helps to mitigate wear of the grinding ball during the friction wear test. The reaction force on the grinding ball influences its sliding, significantly reducing wear intensity. Therefore, as the proportion of SiC increases, both oxidation and adhesive wear decrease to some extent, effectively improving the wear resistance of the cladded coating. As the quantity of SiC added increases, the microsoluble diffusion of SiC powder particles within the cladded coating provides effective protection against frictional wear.

### 3.5. Analysis of Polarization Curve of Cladded Coating

**Figure 13:**
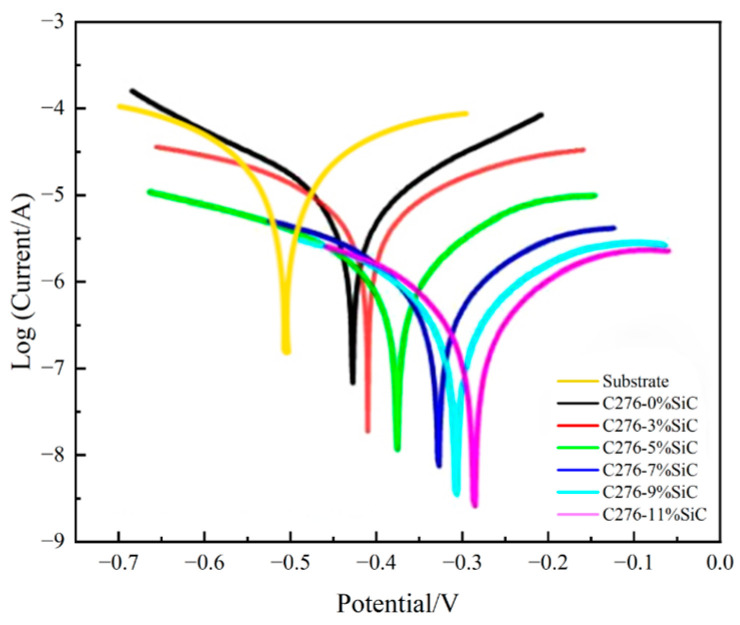
Tafel graph of Hastelloy C276-SiC.

Figure 13 shows the trend of electrochemical corrosion Tafel curves for Hastelloy C276-SiC cladded coatings with varying SiC additions, as well as the 316L substrates in a 3.5 wt% NaCl corrosion solution. Table 5 provides a comparison of the corrosion current density for the 316L substrates and the cladded coatings with different SiC additions. The Tafel graph indicates that both the pure Hastelloy C276 cladded coatings and the SiC-added cladded coatings display an upward trend in corrosion current density and potential within the anode region. However, the rate of increase varies, with the addition of SiC leading to a slower rate of increase. This behavior can be attributed to the relatively complete and dense passivation film of Hastelloy C276, which exhibits good corrosion resistance. As the quantity of SiC is incrementally increased, the corrosion voltage of the molten cladded coating shifts gradually to the right, with recorded values of 418 mV, 409 mV, 385 mV, 337 mV, 315 mV and 289 mV, corresponding to corrosion current density values of 1.377×10⁻^5^ A/cm^2^, 1.138 × 10⁻^5^ A/cm^2^, 9.593 × 10⁻^6^ A/cm², 8.433 × 10^6^ A/cm^2^, 6.884 × 10^6^ A/cm^2^ and 5.543 × 10^6^ A/cm^2^, respectively. The corrosion voltage of the 316L substrate is 512 mV, with a corrosion current density of 1.022 × 10⁻^5^ A/cm^2^. This suggests that as the corrosion potential increases, the corrosion current density decreases, indicating an improvement in corrosion resistance. This phenomenon can be attributed to the diffusivity of SiC powder particles in the cladded coatings, which promotes the formation of a dendritic structure composed of silicon and carbon elements. This, in turn, enhances the uniformity and density of the overall cladded coating structure. Furthermore, the potential of the diffusible SiC powder particles is higher than that of the cladded coatings themselves, allowing them to function similarly to a passivation film on the surface of the cladded coating [31]. An increase in SiC addition corresponds to a rise in the number of dissolved and diffused SiC powder particles within the cladded coatings. These diffusible SiC powder particles have the potential to significantly impair the ductility of the grain structure, leading to a reduction in grain size and an enhancement of the overall microdensity of the cladded coatings. At 11 wt% SiC addition, the concentration of diffusible SiC powder particles in the Hastelloy C276-SiC cladded coating reaches its maximum. The passivation-like film produced by these particles can mitigate the increasing trend of corrosion current density observed in the curve, and the extent of its passivation region reaches its maximum accordingly [32].

### 3.6. Corrosion Behavior Analysis

**Figure 14:**
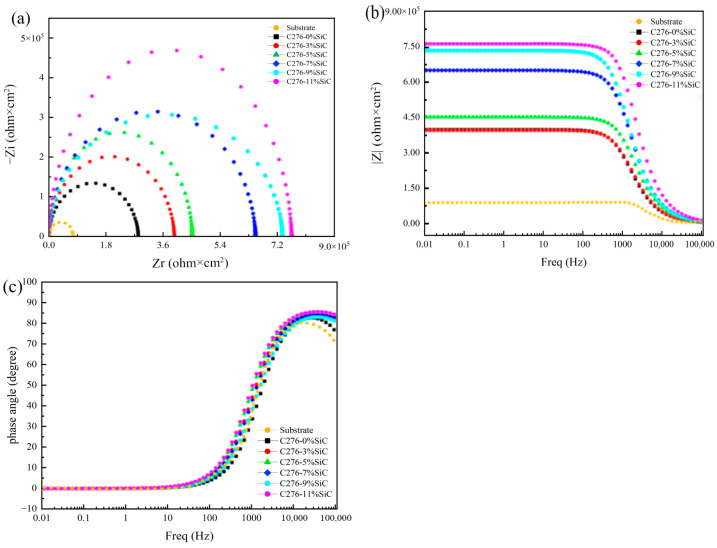
Comparative EIS plots of Hastelloy C276-SiC melt cladded coatings. (**a**) Nyquist diagram; (**b**) Bode impedance modulus diagram; (**c**) Bode phase angle diagram.

**Figure 15:**
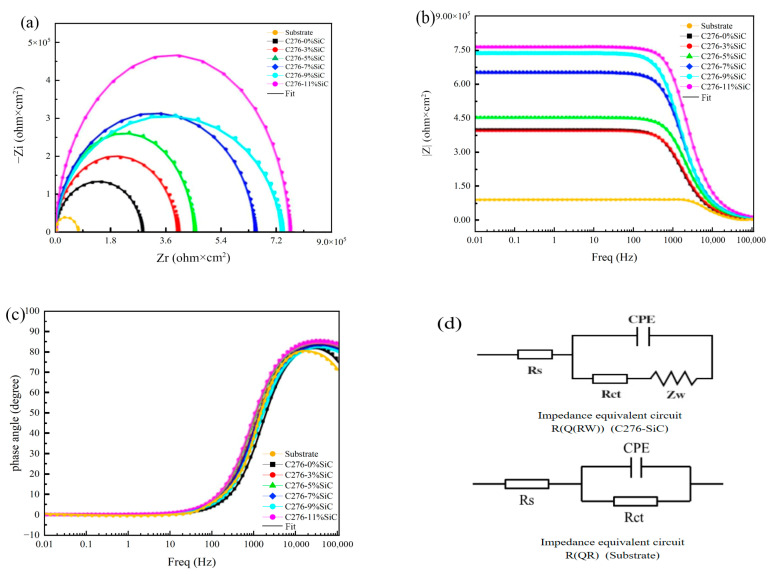
Experimental simulated EIS diagram of Hastelloy C276-SiC fusion cladded coatings. (**a**) Impedance comparison; (**b**) modulus comparison; (**c**) phase angle comparison; (**d**) the equivalent circuit diagram of the Hastelloy C276-SiC cladded coatings and 316L substrates.

The electrochemical AC impedance mapping curves of the C276-SiC cladded coatings, featuring varying SiC additions in a 3.5 wt% NaCl corrosion solution, are presented in Figure 14. The overall test frequency domain for the EIS mapping ranges from 10⁻^2^ Hz to 10^5^ Hz. Figure 15a illustrates the Nyquist mapping of the C276-SiC cladded coatings with different SiC additions, revealing that the shape of the plot comprises semicircular capacitance arcs. As the amount of SiC addition increases, the diffusivity of SiC powder particles within the cladded coating also rises. This enhancement refines the grain size and inhibits the growth of the crystal structure, resulting in a densely distributed microstructure throughout the cladded coating. Additionally, the increased diffusivity of SiC powder particles promotes the formation of an oxide film, which contributes to corrosion resistance. An increase in SiC quantity correlates with a rise in the radius of curvature of the capacitive arc in the EIS diagram. The Bode impedance modulus of the cladded coatings with varying SiC additions is depicted in Figure 15b, while the Bode phase angle for the cladded coating with different SiC additions is shown in Figure 15c. The Bode modulus diagram indicates that the EIS modulus value gradually declines with an increase in frequency. However, when the scanning frequency exceeds 1000 Hz, the Bode modulus value reaches a plateau stage of slow decline, corresponding to the regions adjacent to the larger radius of curvature of the capacitive arc in the EIS impedance diagram. The Bode curves of the EIS modulus values and scanning frequencies demonstrate that the impedance values of the Hastelloy C276-SiC cladded coatings increase with the addition of SiC, indicating an improvement in the corrosion resistance of these coatings. Notably, when the SiC content reaches 11 wt%, the corrosion resistance is optimized. The Bode phase angle diagram reveals that the phase angle values at low and medium frequencies exceed those at high and very low frequencies, suggesting the presence of a peak in the low-frequency region of the curve. Furthermore, to ascertain the number of chemical reactions occurring during the experiment, it is essential to correlate the transformation trend of the EIS phase angle with the actual equivalent circuit fitting results. This approach facilitates a more comprehensive evaluation of the experimental data [33].

Further analysis of the impedance mapping data must be conducted alongside the equivalent circuit parameter values throughout the electrochemical reaction. Figure 15d presents the equivalent circuit diagram of the Hastelloy C276-SiC cladded coatings and 316L substrates. The equivalent R(Q(RW)) circuit is deemed the most suitable for simulating the Hastelloy C276-SiC cladded coatings. As shown in Figure 15, the simulated and actual measured data for the Hastelloy C276-SiC clad coatings under the influence of a 3.5 wt% NaCl corrosion solution are presented, testing the full frequency domain from 10^−2^ to 10^5^ Hz. Figure 15a shows the Nyquist plot comparing the equivalent R(Q(RW)) circuit simulation with the actual test data, while Figure 15b depicts the Bode modulus plot of both the equivalent circuit simulation and the actual test data. Additionally, Figure 15c illustrates the Bode phase angle plot for the same comparison. In these figures, the scattered portion of the plot represents the equivalent R(Q(RW)) circuit simulation data, while the dashed portion denotes the experimental data. The black square scattered data show the highest degree of agreement with the experimental results, while the remaining five simulated data sets also demonstrate a strong alignment with the experimental data and exhibit a trend consistent with a single time constant for the phase angle data. This suggests that a single time constant corresponds to a single parallel circuit, indicating that the electrochemical parameters of the equivalent R(Q(RW)) circuit simulation align well with the actual experimental data for the Hastelloy C276-SiC cladded coatings. For the 316L substrates, the conventional R(QR) simulation circuit was selected.

The parameter values for each component in the equivalent R(Q(RW)) and R(Q(R)) circuits of the Hastelloy C276-SiC cladded coatings and the 316L substrates, obtained through fitting with ZsimpWin 3.20 software, are presented in Table 6. The data indicate that as the amount of SiC increases, the Rct value of the Hastelloy C276-SiC cladded coatings also rises. This suggests that the incorporation of SiC enhances the aforementioned properties. The increased diffusivity of SiC powder particles within the cladded coatings, resulting from the higher quantity of SiC added, effectively inhibits the growth of the grain structure, thereby promoting the formation of a dense and uniformly distributed grain microstructure. Furthermore, the surfaces of the cladded coatings in acidic and alkaline solutions develop a compact passivated oxide film, which contributes to the elevated charge-transfer resistance of the cladded coatings.

For the above optimal equivalent circuit, the electrochemical impedance software was utilized to assess the stability and reliability of the baseline values for the optimal equivalent circuit. As illustrated in Table 7, the base test values for the equivalent R(Q(RW)) and R(Q(R)) circuits of Hastelloy C276-SiC cladding and 316L substrate reveal that the minimum base test value for the 316L substrate is 5.03 × 10^−9^, while the minimum base test value for the five claddings is 6.27 × 10^−7^, with a maximum value of 1.26 × 10^−6^. Notably, the equivalent circuit base for the five coatings is less than 1 × 10^−4^. This indicates that the simulated impedance data aligns well with the experimental results, effectively representing the topology of the EIS test simulation data.

## 4. Conclusions

In this paper, optimal process parameters were employed to prepare a melt clad coating of Hastelloy C276-SiC on stainless steel 316L, incorporating SiC hard phase powder at mass fractions of 0%, 3%, 5%, 7%, 9% and 11%. The major results are summarized as follows:

(1) The incorporation of varying mass fractions of SiC powder resulted in the formation of high-hardness phase components in the coating, including Cr_3_Si and Si_3_C_3_, which enhanced the microhardness and wear resistance of the coating.
(2) With an increase in SiC addition, the diffusion coefficient of SiC powder particles in the cladding layer also increased. This process refined the grain size and inhibited the growth of the crystal structure, leading to the formation of a densely distributed microstructure in the cladding coating. Additionally, the diffusivity of the SiC powder particles facilitated the formation of an oxide film, thereby enhancing the corrosion resistance of the coating.
(3) The average microhardness of the 9 wt% SiC coating was 670.1 HV_0.5_, which was 3.05 times that of the substrate and 1.19 times that of the 0 wt% SiC coating. The measured wear rate was 14.32 × 10^−6^ mm^3^N^−1^·m^−1^, equivalent to 27.35% of the substrate value and 40.74% of the 0 wt% SiC coating. The electrochemical corrosion potential was 315 mV, which was 218 mV higher than that of the substrate and 103 mV higher than that of the 0 wt% SiC coating. The electrochemical charge-transfer resistance was approximately 25 times that of the substrate and 1.26 times that of the 0 wt% SiC coating.

## Figures and Tables

**Table 1:** Elemental composition of stainless steel 316L (wt%).

Element	Fe	Cr	Ni	Mo	Mn	Si	P	C	S
Mass fraction	Bal.	17	13	3	2.5	1.5	0.045	0.04	0.030

**Table 2:** Elemental composition of Hastelloy C276 powder (wt%).

Element	Ni	Mo	Cr	Fe	W	Co	Mn	V
Mass fraction	Bal.	16.5–17	16.3–16.5	6.8–7.0	4.2–4.5	2.0–2.5	0.7–1.0	0.2–0.3

**Table 3:** EDS spot sweep results around SiC particles in the clad layer of 9 wt% SiC.

Point	Ni	Cr	Mo	Si	C	Fe	W
A	48.2	13.1	11.8	9.5	3.1	10.8	3.5
B	48.7	12.2	10.9	9.9	3.3	11.2	3.8
C	-	-	-	65.7	34.3	-	-
D	49.1	11.9	11.5	9.4	2.9	10.9	4.3
E	-	-	-	64.8	35.2	-	-
F	49.2	12.8	10.4	9.1	3.5	11.3	3.7

**Table 4:** Results of EDS point sweep analysis after wear of the molten cladded coating with varying SiC additions (wt%).

Point	Ni	Cr	Mo	Fe	O	Si	C
A	10.1	17.7	1.6	65.6	3.2	0.2	-
B	11.5	16.6	1.4	64.3	4.4	0.6	-
C	51.0	14.2	11.9	11.4	6.1	4.2	1.2
D	48.6	13.7	12.5	12.7	7.2	3.9	1.4
E	48.7	14.7	11.3	11.2	9.3	3.4	1.4
F	46.8	14.2	12.7	11.9	9.1	3.6	1.7
G	48.6	13.6	11.9	10.5	7.2	5.7	2.5
H	51.3	12.9	12.6	9.4	5.8	5.9	2.1
I	43.4	13.5	12.3	12.1	7.4	7.8	3.5
J	44.2	14.1	11.8	11.5	6.4	8.1	3.9
K	40.9	12.8	12.7	10.3	8.7	10.2	4.4
L	40.5	13.4	11.7	11.7	7.3	10.7	4.7
M	37.1	14.3	12.5	9.2	8.9	12.5	5.5
N	38.6	13.8	12.9	10.2	7.5	11.9	5.1

**Table 5:** Comparison of corrosion parameters of Hastelloy C276-SiC clad layers with different SiC additions.

Working Electrode	Ecorr/mv	Icorr/(A·cm^−2^)
Substrate	512	1.022 × 10^−5^
0 wt% SiC	418	1.377 × 10^−5^
3 wt% SiC	409	1.138 × 10^−5^
5 wt% SiC	385	9.593 × 10^−6^
7 wt% SiC	337	8.433 × 10^−6^
9 wt% SiC	315	6.884 × 10^−6^
11 wt% SiC	289	5.543 × 10^−6^

**Table 6:** Equivalent component specific values.

Rs (ohm × cm^2^)		CPE		Rct (ohm × cm^2^) Zw (ohm × cm^2^ × s^−0.5^)	
		Y0 (ohm^−1^ × cm^−2^ × sn) n (0 < n < 1)			
Substrate	5.23	1.89 × 10^−3^	0.74	2.62 × 10^5^	——
0 wt%SiC	28.34	3.53 × 10^−5^	0.97	5.18 × 10^6^	1.68 × 10^−8^
3 wt%SiC	28.98	3.32 × 10^−5^	0.98	5.54 × 10^6^	1.85 × 10^−8^
5 wt%SiC	30.57	3.05 × 10^−5^	0.97	5.98 × 10^6^	2.33 × 10^−8^
7 wt%SiC	34.52	2.82 × 10^−5^	0.98	6.23 × 10^6^	2.67 × 10^−8^
9 wt%SiC	38.97	2.72 × 10^−5^	0.96	6.54 × 10^6^	3.18 × 10^−8^
11 wt%SiC	41.53	2.28 × 10^−5^	0.97	7.53 × 10^6^	3.78 × 10^−8^

**Table 7:** Equivalent circuit chi-square test values.

Working Electrode	Equivalent Circuit	Cardinality
Substrate	Rs(Q(Rct))	5.03 × 10^−9^
0 wt%SiC	Rs(Q(RctW))	6.27 × 10^−7^
3 wt%SiC	Rs(Q(RctW))	8.17 × 10^−7^
5 wt%SiC	Rs(Q(RctW))	1.26 × 10^−6^
7 wt%SiC	Rs(Q(RctW))	1.86 × 10^−6^
9 wt%SiC	Rs(Q(RctW))	4.70 × 10^−6^
11 wt%SiC	Rs(Q(RctW))	7.07 × 10^−7^

## Data Availability

Data are contained within the article.
